# Psychosocial, education, economic factors, decision-making ability, and caries status of mothers of children younger than 6 years in suburban Nigeria

**DOI:** 10.1186/s12903-020-01120-8

**Published:** 2020-05-06

**Authors:** Morenike Oluwatoyin Folayan, Maha El Tantawi, Ayodeji Oginni, Abiola Adeniyi, Michael Alade, Tracy L. Finlayson

**Affiliations:** 1 Obafemi Awolowo University, Ile-Ife, Nigeria; 2 Faculty of Dentistry, Alexandria University, Alexandria, Egypt; 3 Innovative Aid, Abuja, Nigeria; 4 Lagos State University College of Medicine, Lagos, Nigeria; 5 Obafemi Awolowo University Teaching Hospitals’ Complex, Ile-Ife, Nigeria; 6 School of Public Health, San Diego State University, San Diego, CA USA

**Keywords:** Caries, Anxiety, Depression, Parenting stress, Executive dysfunction, Sense of coherence, Fatalism, Social support, Socio-economic status, Decision-making status

## Abstract

**Background:**

Little information is available on the relationship between mothers’ psychosocial profile and caries status, and less information is available on the oral health status and psychosocial status of mothers of young children in Africa. This study examined the association between the psychosocial profile of mothers in Nigeria and their prevalence of caries.

**Methods:**

The prevalence of caries and severe caries (DMFT > 3) in mothers with children 71 months old and younger recruited through a household survey in Ile-Ife, Nigeria, was estimated through clinical examination. The explanatory variables were maternal education, income, decision-making status, and psychosocial status (dental anxiety, general anxiety, depressive symptoms, parenting stress, executive dysfunction, sense of coherence, fatalism and social support). The risk indicators for maternal caries were analyzed with logistic regression.

**Results:**

The prevalence of caries was 3.3%. Twenty (39.2%) of the 51 women with caries had DMFT > 3. Most study participants were 25–34 years old (59.3%), had secondary level education (63.1%), earned N18,000 ($49)-N30000 ($84) per month (42.9%), and can make autonomous decisions about their health care, household purchases, or visits to family/relatives (68.8%). Most women had normal general anxiety (79.9%), low dental anxiety (90.4%), and normal stress (76.4%) levels. Most also had high fatalism (56.6%), perceived moderate social support (81.6%), had normal depressive symptoms (75.9%), low executive dysfunction (55.9%), and high sense of coherence (53.8%). Mothers who had clinically significant levels of stress were twice more likely to have caries than were those whose level of stress was normal (AOR: 2.26; 95%CI: 1.04–4.89; *P* = 0.039). Also, mothers who had high fatalism were less likely to have caries than were those with low fatalism (AOR: 0.40; 95%CI: 0.21–0.75; *P* = 0.004).

**Conclusion:**

High levels of parenting stress was a risk indicator for caries while high fatalism was protective from caries in mothers of children younger than 6-years. Maternal education, income and decision-making ability were not associated with maternal caries. Though the caries prevalence for women with young children was low, the prevalence of severe caries was high and this because of the possible negative effect on their health and wellbeing.

## Background

A woman’s biology - reproductive function, concentrations of sex hormones, and higher percentage of body fat - have health implications [1]. In addition, genderized behaviors, lifestyle, and life experiences often negatively affect women’s access to health care, use of preventive and curative health care services, and the attitudes of medical personnel towards them [1–3]. Substantial changes in health occur during and after a woman becomes a mother, and these changes may have implications for women’s oral health [1].

The health and psychological wellbeing of mothers as autonomous rather than reproductive beings is important. Few studies have evaluated the oral health of women caring for their highly dependent infants, toddlers and preschool children. Most studies focused on women’s oral health during pregnancy [4]. Paying attention to the health needs of mothers of young children improves the mothers’ quality of life and has implications for the health and wellbeing of her peers, family, and the community she is part of [5].

The stressful period of young-child care can cause new oral health challenges for women, resulting from a complex interaction between biological [6, 7], behavioral [8] and psychosocial [9–11] risk factors that are more pronounced during this time. For example, women face increased risk of violence from partners while nursing children, a factor associated with the risk for poor oral hygiene [12], with its corresponding risk for caries [13, 14]. The young-child-care period is also associated with increased risk for financial stress, with increased tendency for mothers to feed on cheap, high-sugar content food [15], a tendency that is also associated with caries development. The risk for postpartum depression [16] may also increase the maternal incidence of caries [17]. Mothers may face negative psychological problems for up to 3 years after childbirth [16].

Women also face emotional stress when they become mothers [18]. Sex differences may exist in parents’ ability to identify and cope with stressors associated with the major life transition of becoming a parent or expanding their families. Studies suggest that women are less able than men to counterbalance the effects of stressors due to differences in their use of personal coping resources such as sense of coherence (https://www.ncbi.nlm.nih.gov/books/NBK435812/). Sense of coherence is a concept from the salutogenic health model that reflects an individual’s global orientation to life and captures resiliency and the ability to cope with problems or stressors [19]. Women can, however, mobilize social support to moderate the effect of stress better than men [20–23]. These psychosocial factors have implications for oral health [24] and are associated with maternal morbidities during the young-child-care period [25–27].

Possible protective factors from caries for women include education, empowerment and psychosocial factors such as executive function, sense of coherence, and social support [28]. Empowered women can make independent decisions about household activities, including access to healthcare, home purchases, and socialization [29]. The ability to maximize health benefits is enhanced by one’s executive function and sense of coherence. Caries prevalence and severity is lower in women with higher education [19]. Higher executive function is associated with improved dental-related functionality [30, 31] and oral hygiene practice [32]. A higher sense of coherence is associated with improved oral health through improved tooth brushing, utilization of dental services, and reduced smoking [33]. Stress, however, impacts negatively on executive function [34].

With increasing evidence of psychosocial risk and protective factors on health and wellbeing, there is need to generate more data on how these affect oral health, including sex-specific data. Little is known about the oral-health profile of women with children who are heavily dependent on them for care – infants, toddlers and preschool children. This study addresses this gap by examining the association between the psychosocial profile of mothers of 6–71-months-old children and their prevalence of caries.

## Methods

This is a secondary analysis of a dataset that was collected to determine the association between maternal psychosocial factors and early childhood caries through a household survey and clinical examinations conducted between December 2018 and January 2019. The study was conducted in sub-urban Ife Central Local Government Area of Osun State, one of the 774 local government areas in Nigeria. Study participants were identified through three-level multi-stage cluster sampling that led to recruitment of 1549 mother-child dyads. Folayan et al. [35] have described the sampling method and data collection in detail.

### Maternal characteristics

Sociodemographic characteristics included the mother’s age at last birthday, categorized as: < 24 years, 25–34 years, 35–44 years, and > 45 years. Information on mothers’ education (no formal education, primary school only, secondary school only, or post-secondary school) and maternal monthly income (≤N18,000 ($49), N18,001–30,000 ($84), N30,001 – N60,000 ($168), and > N60,000 ($168) [36] were collected by self-report as part of the household survey.

### Maternal decision-making

Maternal decision-making ability, measured by the Demographic Health Survey instrument [37], was based on responses to three questions related to the person who usually makes decisions on: (1) her healthcare, (2) large household purchases, and (3) visits to family or relatives. The responses was either ‘yes’ they made the decision, or ‘no’ someone else made the decision.

### Dental anxiety

Dental anxiety refers to responses to dental associated stress [38]. It was measured using the Modified Dental Anxiety Scale, a reliable, valid and popular questionnaire used to assess dental anxiety in adults [39]. This scale, which has been validated for use in Nigeria [40], is a brief, 5-item Likert scale questionnaire. Responses to items are scored as: not anxious =1, slightly anxious = 2, fairly anxious =3, very anxious =4 and extremely anxious =5. Responses are summed to produce a total score ranging from 5 to 25. Scores 19 and above indicate high dental anxiety, while scores lower than 19 indicate low dental anxiety.

### General anxiety

The 7-item Generalized Anxiety Disorder-7 scale was used to measure generalized anxiety disorder [41]. The scale has high sensitivity and specificity and was used in Nigeria with a group of pregnant women [42]. The Generalized Anxiety Disorder − 7 score is calculated by assigning scores of 0, 1, 2, and 3, to the response categories of ‘not at all’, ‘several days’, ‘more than half the days’, and ‘nearly every day’, respectively. For the overall scores, scores 0-4 was classified as normal anxiety, 5-9 as mild anxiety, 10-14 as moderate anxiety, and 15 to 21 as severe anxiety.

### Parenting stress

A six-item version of the Parenting Stress Index measured maternal stress related to parenting, which was used in the Detroit Dental Health Project [43, 44] and validated for use in the Nigerian population [45], was used for the study. Possible scores for each item are 5 = almost always, 4 = often, 3 = sometimes, 2 = rarely, and 1 = never. Higher scores reflect more frequent experiences of stress. Scores below the 80th percentile indicate normal stress; between 81th to 84th percentiles indicate borderline stress, whereas scores greater than 85% are indicative of high levels of parenting stress [46].

### Sense of coherence

Coherence was measured with the standardized and validated 7-point 13-item Sense of Coherence scale [47] adapted and validated for use in Nigeria [48]. The total score ranged from 13 to 91. The maternal sense of coherence score was divided into lower than the median (low coherence) and equal to and greater than the median (high coherence) score. The median score was 67.

### Depressive symptoms

The Centre for Epidemiologic Studies and Depression Scale developed by Radloff [49] was used to determine the level of depressive symptoms. The scale was validated for use in Nigeria [50]. The 20 items are scored on a scale from 0 to 3, depending on the frequency of symptoms. Symptoms occurring rarely or less than once per day were rated 0; those occurring for some or a little of the time (such as for 1–2 days) were rated 1; occasionally or a moderate amount of time (as for 3–4 days) were rated 2; and those occurring most or all the time (as for 5–7 days) were rated 3. Some questions (numbers 4, 8, 12 and 16) were reverse-scored. Total scores less than 15 indicated no depressive symptoms; scores from 15 to 21 indicated mild to moderate depressive symptoms; and scores over 21 indicated major depressive symptoms.

### Executive dysfunction

The 20-item Modified Dysexecutive questionnaire is a three-factor questionnaire designed to assess everyday changes to cognition, emotion, and behavior associated with executive dysfunction [51]. Each of the 20 statements was read to participants, and they were asked to rate them on a 5-point scale (0–4) ranging from ‘Never’ to ‘Very often’, with higher scores indicating more problems. A sample question is ‘I have problem understanding what other people mean unless they keep things simple and straightforward’. Possible scores range from 0 to 80, with higher scores indicating worse dysfunction. The instrument was validated for use in Nigeria [52]. For this study, executive dysfunction was divided into low executive dysfunction (scores lower than the median) and high executive dysfunction (scores equal to and greater than the median). The median score was 32.

### Fatalism

The Powe Fatalism Inventory [53], modified for use to measure maternal fatalistic beliefs about early childhood caries [54], was used to measure perception of fatalism. Responses to the nine questions ranged from ‘strongly agree’, ‘somewhat agree’, ‘neither agree nor disagree’, ‘somewhat disagree’ to ‘strongly disagree’ on a Likert-like scale with scores from 5 to 1, respectively. The possible scores range from 9 to 45. Content validation was done by pre-testing the instrument in a group of 50 randomly selected mothers from Ife-East Local Government Area for comprehensibility, understanding, and cultural appropriateness of the content. The instrument was validated for use in Nigeria [52]. The median score was 25.

### Social support

The Multidimensional Scale of Perceived Social Support was used to measure emotional support from three sources: family, friends and significant others [55]. It is a 12-item 7-point scale. The total score is obtained by summing the points across all 12 items and dividing by 12. Scores ranging from 1 to 2.9 indicate low support; a score of 3 to 5 indicates moderate support; and a score from 5.1 to 7 indicates high support. The scale was validated for used in Nigeria to measure social support among adolescents [56].

### Maternal caries status

Mothers were also examined under natural light sitting on a chair. Caries was determined using the Decayed-Missing-Filled teeth (DMFT) index as recommended by the World Health Organization [57]. The DMFT score was obtained by adding the D, M and F scores for each mother. Caries was considered present when the DMFT score was greater than 0 and absent when the DMFT was 0. The DMFT equal to or greater than 3 was classified as severe caries [58]. The inter-examiner and intra-examiner agreement Cohen’s kappa coefficient values of the five dentists who conducted the oral examination were higher than 0.80.

### Data analysis

Descriptive analysis was conducted to determine the proportion of women with different attributes based on the study psychosocial (dental anxiety, general anxiety, depressive symptoms, parenting stress, executive dysfunction, sense of coherence, fatalism and social support), socioeconomic (education, income, decision-making status) and caries status (DMFT) variables. Bivariate analysis was conducted to determine the association between the psychosocial and socioeconomic variables and the prevalence of maternal caries. Chi-square test was used for bivariate analysis, and Fisher’s exact test was conducted when 25% of the cells had expected count less than 5. Logistic regression was conducted to determine the psychosocial and socioeconomic risk indicators for maternal caries controlling for the confounding effect of maternal age. The estimated coefficients, expressed as adjusted odds ratios (AOR) and their 95% confidence intervals, were calculated. Statistical analyses were conducted with Intercooled STATA (release 15) for windows. Statistical significance was inferred at *p* < 0.05.

### Ethics approval

Ethical approval for the study was obtained from the Obafemi Awolowo University Teaching Hospitals Complex Health Research Ethics Committee (IRB/IEC/0004553).

## Results

Table 1 lists the socio-demographic characteristics of the study participants. The age range for the mothers was 16–55 years, with mean (standard deviation or SD) age of 31.7 (5.8) years. Most (59.3%) were 25–34 years, had secondary education (63.1%) and earned N18,000 ($49) -N30000 ($84) per month (42.9%). Most participants (68.8%) reported being able to make the three types of decisions autonomously - health care, household purchases, and visits to family and relatives.

**Table 1:** Characteristics of mothers of children 71-months-old and younger in Ile-Ife, Nigeria [*N* = 1549]

Variables	*N* = 1549	
	n	%
**Age**		
≤ 24 years	129	8.3
25–34 years	919	59.3
35–44 years	459	29.6
≥ 45 years	42	2.7
**Education status**		
None	23	1.5
Primary only	115	7.4
Secondary only	977	63.1
Above secondary	434	28.0
**Income status**		
< N18,000 per month	422	27.2
N18,000 – N30,000 per month	665	42.9
N30,001 – N60,000 per month	417	26.9
> N60,000 per month	45	2.9
**Decision-making status**		
Someone else decides on access to health care	394	25.4
Someone else decides on household purchases	322	20.8
Someone else decides on visits to family/relatives	335	21.6
**Decision-taking status**		
Can make the three decisions	1065	68.8
Can make one of three decisions	170	11.0
Can make two of three decisions	61	3.9
Can’t make any decision	253	16.3

Of the 1549 mothers recruited for the study, only 51 (3.3%) had caries. DMFT ranged from 0 to 13 with a mean (SD) of 0.10 (0.76). Of the 51 mothers with caries, 20 (39.2%) had a DMFT > 3. All the carious lesions identified were untreated decayed lesions.

Table 2 highlights the results of the test of association between the psychosocial status of the mother and the prevalence of caries. The only psychosocial factor significantly associated with the prevalence of caries was fatalism: more mothers with low fatalism had caries (*p* = 0.005).

**Table 2:** Bivariate association between the characteristics of mothers of children 71-months-old and younger in Ile-Ife, Nigeria and their prevalence of caries (*N* = 1549)

Variables	Caries status				*P* value
	Absent (*N* = 1498)		Present (*N* = 51)		
	N	%	N	%	
**Age**					
≤ 24 years	125	96.9	4	3.1	0.113
25–34 years	887	96.5	32	3.5	
35–44 years	448	97.6	11	2.4	
≥ 45 years	38	90.5	4	9.5	
**Education status**					
None	21	91.3	2	8.7	0.090
Primary only	109	94.8	6	5.2	
Secondary only	943	96.5	34	3.5	
Above secondary	425	97.9	9	2.1	
**Income status**					
< N18,000 per month	405	96.0	17	4.0	0.494
N18,000 – N30,000 per month	648	97.4	17	2.6	
N30,001 – N60,000 per month	401	96.2	16	3.8	
> N60,000 per month	44	97.8	1	2.2	
**Decision-making status**					
*Someone else decides on access to health care*					
No	1119	96.9	36	3.1	0.322
Yes	378	95.9	16	4.1	
*Someone else decides on household purchases*					
No	1188	96.8	39	3.2	0.400
Yes	310	96.3	12	3.7	
*Someone else decides on visits to family/relatives*					
No	1175	96.8	39	3.2	0.496
Yes	323	96.4	12	3.6	
**Decision-taking status**					
Can make the three decisions	1031	96.8	34	3.2	0.457
Can make one of three decisions	166	97.7	4	2.4	
Can make two of three decisions	57	93.4	4	6.6	
Can’t make any decision	244	96.4	9	3.6	
**General anxiety**					
Normal	1200	96.9	38	3.1	0.416
Mild	234	96.3	9	3.7	
oderate to severe	64	94.1	4	5.9	
**Dental anxiety**					
Low	1354	96.7	46	3.3	0.964
High	144	96.6	5	3.4	
**Parenting stress**					
Normal	1150	97.1	34	2.9	0.198
Borderline	35	97.2	1	2.8	
High	313	95.1	16	4.9	
**Fatalism**					
Low	640	95.2	32	4.8	0.005
High	858	97.8	19	2.2	
**Social support**					
Low	28	93.3	2	6.7	0.202
Moderate	1227	97.1	37	2.9	
High	243	95.3	12	4.7	
**Depressive symptoms**					
Normal	1135	96.6	40	3.4	0.908
Mild-moderate	201	97.1	6	2.9	
Major	162	97.0	5	3.0	
**Executive dysfunction**					
Low	841	97.1	25	2.9	0.314
High	657	96.2	26	3.8	
**Sense of coherence**					
Low	689	96.2	27	3.8	0.328
High	809	97.1	24	2.9	

Table 3 highlights the outcome of the logistic regression analysis. The Pseudo *R*^*2*^ estimate indicates that about 8% of the variance in mothers who had or did not have caries could be explained by the combination of the independent variables in the model. Mothers who had high levels of stress were twice as likely to have caries as were those whose level of stress was normal (AOR: 2.26; 95% CI: 1.04–4.89; *p* = 0.039). Also, mothers who had high fatalistic beliefs were less likely to have caries than were those who had low fatalistic beliefs (AOR: 0.40; 95%CI: 0.21–0.75; *p* = 0.004).

**Table 3:** Logistic regression to determine predictors of caries in mothers of children 71-months-old and younger in Ile-Ife, Nigeria [*N* = 1539]

Variables	Adjusted Odds Ratio	95% confidence interval	*p*-value
**Age**			
≤ 24 years	1.00	–	–
25–34 years	1.56	0.51—4.75	0.432
35–44 years	1.04	0.30—3.53	0.955
≥ 45 years	3.57	0.78—16.32	0.101
**Education status**			
None	1.00	–	–
Primary only	0.76	0.12—4.70	0.765
Secondary only	0.48	0.09—2.47	0.377
Above secondary	0.26	0.04—1.56	0.140
**Income status**			
< N18,000 per month	1.00	–	–
N18,000 – N30,000 per month	0.72	0.34—1.51	0.386
N30,001 – N60,000 per month	1.05	0.46—2.37	0.911
> N60,000 per month	0.65	0.08—5.53	0.694
**Decision-making status**			
*Someone else decides on access to health care*			
No	1.00	–	–
Yes	1.62	0.50—5.25	0.421
*Someone else decides on household purchases*			
No	1.00	–	–
Yes	0.99	0.28—3.46	0.982
*Someone else decides on visits to family/relatives*			
No	1.00	–	–
Yes	0.95	0.32—2.87	0.928
**Decision-taking status**			
Can make the three decisions	1.00	–	–
Can make one of three decisions	0.49	0.14—1.68	0.257
Can make two of three decisions	2.06	0.62—6.82	0.239
Can’t make any decision^a^	–	–	–
**General anxiety**			
Normal	1.00	–	–
Mild	0.49	0.14—1.68	0.257
Moderate to severe	2.51	0.77—8.12	0.126
**Dental anxiety**			
Low	1.00	–	–
High	0.88	0.32—2.39	0.802
**Parenting stress**			
Normal	1.00	–	–
Borderline	1.08	0.13—8.63	0.944
High	2.26	1.04—4.89	0.039
**Fatalism**			
Low	1.00	–	–
High	0.40	0.21—0.75	0.004
**Social support**			
Low	1.00	–	–
Moderate	0.67	0.13—3.41	0.634
High	1.47	0.26—8.44	0.666
**Depressive symptoms**			
Normal	1.00	–	–
Mild-moderate	0.62	0.23—1.69	0.347
Major	0.53	0.17—1.62	0.264
**Executive dysfunction**			
Low	1.00	–	–
High	1.32	0.70—2.48	0.387
**Sense of coherence**			
Low	1.00	–	–
High	0.59	0.28—1.23	0.158

^a^No estimate was generated

## Discussion

The study findings indicate that parenting stress was a significant risk indicator for caries in mothers with children less than 71 months old, and high fatalistic beliefs were a protective factor. Many reasons can be proffered for the increased risk for caries in mothers who experience parenting stress. All the mothers in this sample had young children who are dependent on their caregivers for all their needs. Younger infants and toddlers have constant needs and little ability to communicate those needs or to independently meet them. It would not be unusual for mothers to report parenting demands as highly stressful, particularly if they are first-time mothers who are navigating this transition for the first time. Even experienced mothers may have parenting stress, and many prioritize their children’s needs over their own [59]. This allocation can include change in feeding behavior of stressed individuals – stressed individuals eat more hyper-palatable (high fat, high sugar) food [60], which increases the risk for obesity and caries [61, 62]. Stress also increases the risk for poor oral hygiene; a risk factor for caries [63].

The inverse relationship between the risk for caries and high fatalism is less clear. Fatalism is associated with reluctance to participate in health-promotion programs and health-care utilization because of the belief that participation will have no positive impact and that poor health outcomes are inevitable [64–67]. However, the combination of fatalism, religiosity and race/ethnicity may affect the way that fatalism operates due to differences in the locus of control beliefs (being more oriented to internal or external control over events and outcomes) [68, 69]. Nigerians, like most Africans, endorse religious fatalism [70, 71], and religious fatalism is not an inhibitor to healthy behaviors, nor is it disempowering [72, 73]. Fatalism is, however, a complicated construct that is more a coping response to illness and acceptance of what is beyond individual control than an inhibitor of healthy behaviors [74]. This study finding seems to indicate that for African women with young children, high fatalism, which may be tied to their religiosity, reduces their risk for caries. This study did not measure religiosity. This pathway for how maternal fatalism results in reduced risk for caries needs to be studied in-depth using qualitative methods in the future.

In the present study, the prevalence of caries experience among mothers was low and mostly based on untreated caries. Untreated caries increases the risk for new lesions [74] and the count of *Streptococcus mutans,* which increases the risk of transmission of the organism to children and subsequently their caries risk [75]. Also, the failure of mothers to seek treatment for dental caries likely indicates lower chances of the child utilizing dental preventive care. Despite the overall low caries prevalence, there is a subset of the population where caries problems persist. It is important for health policy planners to study the problems associated with caries in this subset of the population in comparison to the other health needs of mothers and their children. A strategic approach of addressing this caries related problem may be to routinely provide oral health checkups for mothers during pregnancy, and when they visit the clinics for their child health care. This may offer a cost effective healthcare systems dependent approach to manage caries in mothers with young children in resource-limited settings such as those in most African countries.

We found that education, income, and decision-making ability were not associated with caries in mothers of young children in the study population. This could be due to the relative homogeneity in this sample, as most women reported similar levels of educational attainment, modest income and low empowerment. Education and income are social determinants of health that are difficult to modify without substantial policy change. Cultural changes are, however, needed to increase empowerment of women for household decision-making in communities like Nigeria, which are highly patriarchal.

We found no association between the prevalence of caries and mothers’ dental anxiety, general anxiety, depressive symptoms, executive dysfunction, sense of coherence, or social support. This agrees with other studies indicating that adult onset of dental anxiety [76] and general anxiety [77] were not associated with increased risk for caries. In contrast, previous studies showed that depression increases the risk for caries [77], and post-partum depression increases the risk for early childhood caries in young children [78]. The literature on maternal oral health during child-care is sparse, which likely indicates that this subject has not been sufficiently and critically studied. The low caries prevalence in the study population may also mask the effect of these factors on caries.

The study has strengths: First, it used validated scales to assess a wide range of important psychosocial factors. Many of these measures pertain to complex perceptions and constructs that are not unidimensional, that may relate/interact with each other, and that may change over time, so the use of validated scales is essential. Second, psychosocial measures are often not included in oral health research, and when they are, the results have been mixed and the mechanisms by which stressors and resource factors operate are not defined [79–81]. This study has added information about the association between psychosocial factors and caries in an African population. Third, the study highlights the need for actions to address the caries risk of women who have young children as caries may negatively affect their health and wellbeing and the oral health of their children [82]. Fourth, the study reveals that it may be important that mothers of children 71 months of age and younger who seek care for caries, be screened for stress and appropriately managed because this psychosocial factor has implications beyond the immediate oral health of women.

The study also has limitations: First, the design was cross-sectional, so causal links cannot be made. Also because of the design, it cannot be determined if having young children increases the risk of mothers having new caries lesions or if the measured psychosocial status was induced by childcare. Second, the study sample included mothers beyond the traditional postpartum period, and the stressors and resources studied may operate differently in the immediate postpartum period following a child’s birth. Third, the findings may not be generalizable to all Nigerian mothers of young children or to mothers in other countries.

## Conclusion

The prevalence of caries in mothers with children 71 months old and younger in a Nigerian population was 3.3%. High levels of parenting stress is a risk indicator for caries in mothers. High fatalism may be protective of caries. Other psychosocial factors (dental anxiety, general anxiety, depression, executive dysfunction, sense of coherence, and social support) were not associated with caries and maternal education, income and decision-making ability were not risk indicators for caries in the women in our study. The caries risk of women who have young children needs attention because of the possible negative effect on their health and wellbeing and increase the risk of early childhood caries in their children. Longitudinal and qualitative studies are needed to further explore these findings.

## Data Availability

Study-related data and materials and data collection tool are accessible on request from the lead author.

## Abbreviations

AOR: Adjusted Odds Ratio
CI: Confidence Interval
DMFT: Decayed Filled Missing Tooth
GAD: Generalized Anxiety Disorder
SD: Standard Deviation
